# Neuronify: An Educational Simulator for Neural Circuits

**DOI:** 10.1523/ENEURO.0022-17.2017

**Published:** 2017-03-17

**Authors:** Svenn-Arne Dragly, Milad Hobbi Mobarhan, Andreas Våvang Solbrå, Simen Tennøe, Anders Hafreager, Anders Malthe-Sørenssen, Marianne Fyhn, Torkel Hafting, Gaute T. Einevoll

**Affiliations:** 1 Centre for Integrative Neuroplasticity, University of Oslo, 0316 Oslo, Norway; 2 Department of Physics, University of Oslo, 0316 Oslo, Norway; 3 Department of Biosciences, University of Oslo, 0316 Oslo, Norway; 4 Department of Informatics, University of Oslo, 0316 Oslo, Norway; 5 Institute of Basic Medical Sciences, University of Oslo, 0316 Oslo, Norway; 6 Faculty of Science and Technology, Norwegian University of Life Sciences, 1432 Ås, Norway

**Keywords:** app, modeling, neural networks, software, teaching

## Abstract

Educational software (apps) can improve science education by providing an interactive way of learning about complicated topics that are hard to explain with text and static illustrations. However, few educational apps are available for simulation of neural networks. Here, we describe an educational app, Neuronify, allowing the user to easily create and explore neural networks in a plug-and-play simulation environment. The user can pick network elements with adjustable parameters from a menu, i.e., synaptically connected neurons modelled as integrate-and-fire neurons and various stimulators (current sources, spike generators, visual, and touch) and recording devices (voltmeter, spike detector, and loudspeaker). We aim to provide a low entry point to simulation-based neuroscience by allowing students with no programming experience to create and simulate neural networks. To facilitate the use of Neuronify in teaching, a set of premade common network motifs is provided, performing functions such as input summation, gain control by inhibition, and detection of direction of stimulus movement. Neuronify is developed in C++ and QML using the cross-platform application framework Qt and runs on smart phones (Android, iOS) and tablet computers as well personal computers (Windows, Mac, Linux).

## Significance Statement

Neuronify, a new educational software application (app) providing an interactive way of learning about neural networks, is described. Neuronify allows students with no programming experience to easily build and explore networks in a plug-and-play manner picking network elements (neurons, stimulators, recording devices) from a menu. The app is based on the commonly used integrate-and-fire type model neuron and has adjustable neuronal and synaptic parameters. To facilitate teaching, Neuronify comes with premade network motifs performing functions such as input summation, gain control by inhibition, and detection of direction of stimulus movement. Neuronify is available from http://ovilab.net/neuronify for smart phones (Android, iOS), tablet computers, and personal computers (Windows, Mac, Linux).

## Introduction

Over the past decades, simulation and modeling of neurons have become essential tools in neuroscience. Although modern software continues to make modeling more accessible (Gleeson et al., 2007), some programming experience is often required. This makes it difficult for students to explore computational models early in their education. However, educational software applications (apps) allow interaction with computational models without knowledge of programming.

Educational apps have become more common in science, and while such apps exist in many fields, few intuitive and accessible apps have been made for simulating neural networks both on smart phones, tablet computers, and personal computers. Previous efforts have, however, made computational models more accessible. Neurons in Action (Stuart, 2008; Moore and Stuart, 2017) and Neuromembrane (Ali et al., 2017) are examples of interactive tutorials for exploring properties of excitable membranes and neurons, while Emergent (Aisa et al., 2008), SimBrain (Tosi and Yoshimi, 2016), and SpineCreator (Cope et al., 2017) are examples of graphical applications where students can design and analyze neural networks on personal desktop computers.

We have developed an educational app, Neuronify, that goes beyond previous educational tools in that it enables students to easily create neural networks in a plug-and-play manner and is available also on smart phones and tablet computers. It thus provides a new low-threshold entry point to simulation-based neuroscience for students with no programming experience.

With Neuronify, we aim to improve teaching of neural networks and circuits to neuroscience students, by a combination of demonstrating existing circuits, challenging the user with exercises and allowing the user to explore the environment freely. The app makes it easy to teach about complicated network behavior such as direction selectivity based on lateral inhibition (Barlow and Levick, 1965; Fried et al., 2005), where stimuli moving in the non-preferred direction is prevented from inducing firing in the output neuron by a strong, temporally coordinated, inhibitory input volley set up by a tailor-made neural circuit. In the teaching of neuroscience courses, lateral inhibition is one of many examples of networks that are hard to explain with static illustrations. By live visualization of the network, it is possible to explain the process thoroughly by showing how the process works in slow motion. We show how this example is implemented in Neuronify in the Results section (Direction-selective network).

**Figure 1.**
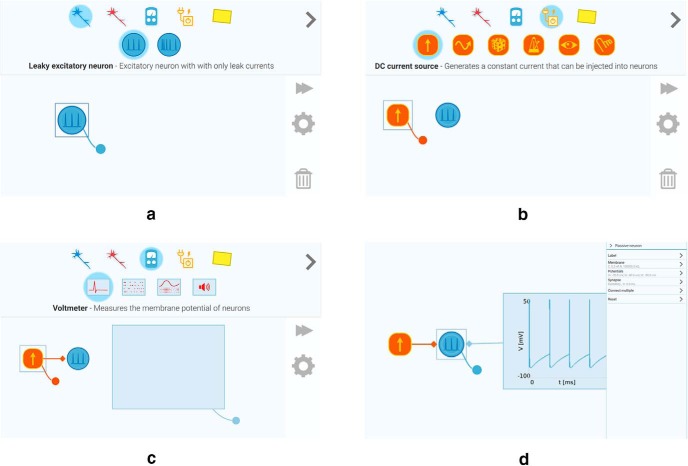
Step-by-step illustration of how to build a simple neural circuit in Neuronify. ***A***, A neuron is added to the canvas by dragging it from the creation menu. ***B***, A DC current source is added and connected to the neuron by dragging the DC current source connection handle onto the neuron. ***C***, A voltmeter is added and connected to the neuron by dragging the voltmeter connection handle onto the neuron. ***D***, The properties of neurons and other items can be changed in the properties panel.

To build and explore neural networks in the app, you drag and drop neurons onto the app’s workspace. The neurons are connected by pulling synapses between them. Once connected, the neurons send a signal to each other whenever they spike. Neurons can also be driven by current sources, spikes generator, and touch and visual input provided via the smart phone, tablet, or computer peripherals. The neurons can be probed by various type of sensors such as voltmeters and spike detectors, and the latter can be forwarded to the loudspeaker. A step-by-step illustration on building a simple circuit is shown in Figure 1. The user can explore how changing the properties of a single cell leads to changes in entire networks. Additionally, the app comes with several premade simulations of common neural network motifs performing functions such as input summation, gain control by inhibition, and detection of direction of stimulus movement. Neuronify runs on smart phones (Android, iOS), tablet computers, and personal computers (Windows, Mac, Linux).

The article is structured as follows. We describe the circuit elements, the integrate-and-fire model, and go into detail on how the app is implemented in Materials and Methods. Then, we show some examples on how Neuronify can be used in Results. Lastly, we make some concluding remarks and discuss future prospects in Discussion.

## Materials and Methods

In this section, we present the Neuronify workspace, the available circuit elements, and technical aspects.

### Workspace

**Figure 2.**
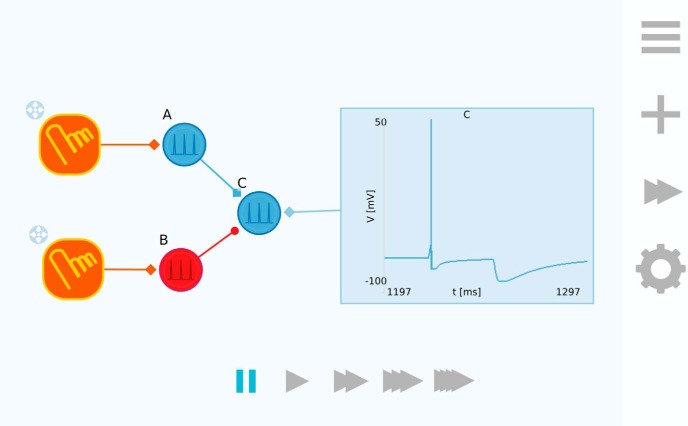
Neuronify workspace. Here, a simulation has been loaded where two touch input sensors are connected to one excitatory neuron (A) and one inhibitory neuron (B). Neuron C is connected to a voltmeter that plots the membrane potential as described by the integrate-and-fire model. This network can be used to illustrate how neuron B can inhibit neuron C so that when neuron A fires shortly after, A may not be able to excite neuron C beyond its threshold potential. Activating neuron A results in a spike in neuron C (first spike in the figure). However, if neuron B is activated first and then neuron A shortly after, neuron C is not excited beyond its threshold potential. To the right we see the toolbar that overlays the workspace and at the bottom we see the playback controls.

The workspace acts as a canvas and is where we expect the user to spend the most time in Neuronify. A simple example network is shown in Figure 2. The circuit elements can be dragged into the workspace and connected to each other.

**Figure 3.**
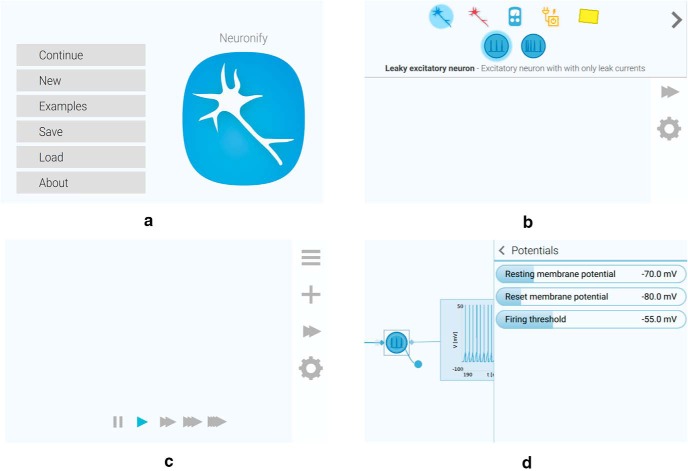
Menus in Neuronify. ***A***, Main menu. ***B***, Creation menu. ***C***, Playback controls. ***D***, Properties panel.

The workspace is overlaid by a toolbar that contains buttons that activates the (from top to bottom) main menu, the creation menu, the playback controls, and the properties panel. All these menus are seen in Figure 3.

The main menu (Fig. 3*A*) is where the user can choose between a new simulation, existing simulations, or save and load own simulations. The creation menu (Fig. 3*B*) is where all the items are found. To add the items to the workspace, the user drags them from the creation menu and drops them onto the workspace. The different items are described in the subsections below.

The playback menu (Fig. 3*C*) allows the user to change the playback speed of the simulation. It ranges from ∼5 ms simulated per 1 s in real time to 50 ms simulated per 1 s in real time. No matter which playback speed is chosen, the temporal resolution of the simulation, however, remains the same. This means that an increase in the playback speed results in a higher computational load for the device running the app.

The properties panel (Fig. 3*D*) is used to modify the properties of items and connections. This includes properties such as cell membrane resistance, current-source output, and synaptic delay, to name a few. Neurons can also be assigned labels that are used to identify them in other contexts, such as in the voltmeter plot labels (see below, Voltmeter).

### Neurons

In the current version of Neuronify, two types of integrate-and-fire neurons are available: leaky and adaptive. Further, both types can be either excitatory or inhibitory.

#### Leaky integrate-and-fire neurons

The integrate-and-fire model (see, e.g., Sterratt et al., 2011) is the most commonly used spiking neuron model and is a standard part of the curriculum in neuroscience courses with a computational component. It has been demonstrated to be very useful for understanding how neurons process information (Burkitt, 2006). Each neuron is modeled as a point neuron, i.e., the soma and dendrites are assumed to be equipotential. The membrane potential describes the state of the neuron. Without any external input, the membrane potential decays like an RC electric circuit toward the resting membrane potential *V_r_*, which is why the neuron is called “leaky.”

A spike (action potential) is generated when the membrane potential reaches the threshold potential *V*_thres_. When the neuron generates a spike, the membrane potential is reset to its initial potential *V*_reset_, which is often defined to be equal to the resting potential *V_r_*. After the spike, the membrane potential is fixed to *V*_reset_ for an absolute refractory period *τ_r_*. Otherwise, the dynamics of the neuron’s membrane potential is described as (Burkitt, 2006):

(1) $$C_{m}\frac{\text{d}V}{\text{d}t}=I_{\text{leak}}+I_{\text{syn}}+I_{\text{inj}}.$$

Here, *C_m_* is the membrane capacitance, *I*_leak_ is the current that drives the decay toward the resting potential, *I*_syn_ is the sum of synaptic input currents, and *I*_inj_ is the sum of injected currents.

With no synaptic inputs or injected currents, Equation 1 is defined to be equivalent to the equation for an electrical circuit with a resistor and capacitor in parallel (RC circuit). The leak current is therefore defined as:

(2) $$I_{\text{leak}}=-\frac{1}{R_{m}}(V-V_{r}).$$

Here, *V_r_* is the resting potential and *R_m_* is the resistance of the membrane. The membrane time constant is given by $\tau_{m}=R_{m}C_{m}$. Note that both *R_m_* and *C_m_* are assumed to be constant.

**Figure 4.**
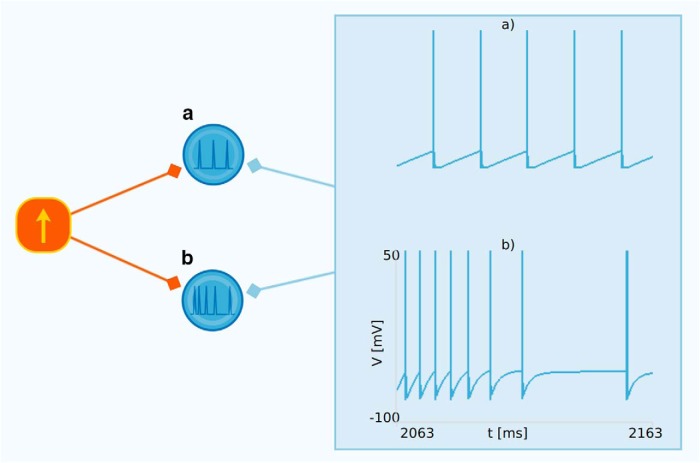
***A***, Leaky integrate-and-fire neuron. The membrane potential of a leaky neuron is shown as plotted by the voltmeter item. As can be seen, the membrane potential increases until it reaches its threshold value and is immediately reset to the initial potential. The spike itself is overlayed as a vertical line for illustrative purposes and is not explicitly included in the dynamics of the membrane potential. ***B***, Adaptive leaky integrate-and-fire neuron. The membrane potential of an adaptive neuron as plotted by the voltmeter item. This neuron receives input from the same DC current source. The interval between each spike of the adaptive neuron increases due to the an additional hyperpolarizing current, which grows for each spike and decays between the spikes.

In Neuronify, the membrane resistance (*R_m_*), membrane capacitance (*C_m_*), resting potential (*V_r_*), reset potential (*V*_reset_), firing threshold (*V*_thres_), refractory period (*τ_r_*), and synapse type (excitatory or inhibitory) can be changed in the properties panel. A figure of the leaky neuron spiking is shown in Figure 4*A*.

#### Adaptive leaky integrate-and-fire neurons

In many neurons, the firing rate decreases when they receive a sustained input. The standard leaky integrate-and-fire model is not able to reproduce such behavior but can easily be extended to incorporate adaptation (Brette and Gerstner, 2005). Here, this is done by adding an additional hyperpolarizing current *I*_adapt_ to Equation 1. The adaptive conductance of this current is incremented by an amount $\Delta g_{\text{adapt}}$, whenever the neuron spikes (Koch, 1999; Latham et al., 2000). In between spikes, the adaptive conductance decays with a time constant $\tau_{\text{adapt}}$:

(3) $$\frac{\text{d}g_{\text{adapt}}}{\text{d}t}=-\frac{g_{\text{adapt}}}{\tau_{\text{adapt}}},$$

(4) $$I_{\text{adapt}}=g_{\text{adapt}}(V-V_{r}).$$

As the activity in a neuron increases, the adaptive current will also increase due to the growing adapting conductance, making it harder for the cell to fire.

The adaptation time constant ($\tau_{\text{adapt}}$), adaptation conductance (*g*_adapt_), and the synapse type (excitatory or inhibitory) can be changed in the properties panel. The spiking of an adaptive neuron receiving a regular spiking input is seen in Figure 4*B*.

### Synapses

The synaptic input to an integrate-and-fire neuron can be modeled in at least two ways: as a conductance-based synapse or a current-based synapse (Sterratt et al., 2011). With a conductance-based synapse model where the current depends on the difference between the membrane potentials and the reversal potential of the synapse, the maximum current is limited. For current-based synapses, there are no such inherent limitations, and the neuron’s membrane potential may increase or decrease without limits. The current-based synapse makes the model easier to analyze and faster to simulate. In Neuronify, connecting two neurons will create a current-based synapse. Current-based synapses are also created when connecting regular spike generators, irregular spike generators, or visual inputs to neurons.

The time course of a synaptic input current is described by a decaying exponential function:

(5) $$I_{\text{syn}}=\{\begin{array}{ll} {\overset{¯}{I}}_{\text{syn}}\exp\left(-\frac{t-t_{s}}{\tau_{\text{syn}}}\right) & \text{for }t\geq t_{s}\\ 0 & \text{for }t<t_{s}. \end{array}$$

Here, ${\overset{¯}{I}}_{\text{syn}}$ is the maximum current (Sterratt et al., 2011).

The maximum current, the synaptic time constant, and the signal delay can be adjusted in the properties panel. Since with current based synapses we risk that the membrane potential goes far beyond the reversal potentials of the involved ions, we limit the membrane potential to be within the range -90 to 60 mV. These are the reversal potentials for K^+^ and Na^+^, respectively. These limits can be modified or disabled by the user.

### Neuron activators

Neuronify comes with several neuron activators that can be used to drive neural circuits, including DC and AC current generators, regular spike generators, and irregular (random) spike generators. Input from the user can be used in the form of touch or visual input.

#### DC and AC current sources

The DC current source is an item that, when connected to a neuron, injects constant current into the neuron. The AC current source injects an alternating current with the form of a sine wave. The amount of injected current can be adjusted by the user. The frequency of the sine-wave current can also be adjusted for the AC source.

#### Regular spike generators

The regular spike generator produces spikes with a constant firing rate. Connected neurons will experience these spikes as if they were received as synaptic input from a regularly firing neuron. Connecting a regular spike generator to a neuron creates a current-based synapse, with properties that can be modified as described above in Synapses. The generator can produce both excitatory and inhibitory output, i.e., mimicking afferent inputs both from excitatory and inhibitory neurons.

#### Irregular spike generators

The irregular spike generator produces a train of randomly timed spikes with an average firing rate specified by the user. The spikes follow a homogeneous Poisson process (Dayan and Abbott, 2005). For every time step of the simulator, there is a constant probability that the generator will produce a spike. As for the regular spike generator, the generator can produce excitatory or inhibitory spikes, i.e., mimicking afferent inputs both from excitatory and inhibitory neurons. The synaptic connection is the same as for the regular spike generator.

#### Touch activator

A touch activator makes connected neurons fire when activated. On mobile devices with a touch screen, the sensor is activated by touching it. On desktop versions of the app, the sensor is activated by left-clicking on it with the mouse.

#### Visual input

**Figure 5.**
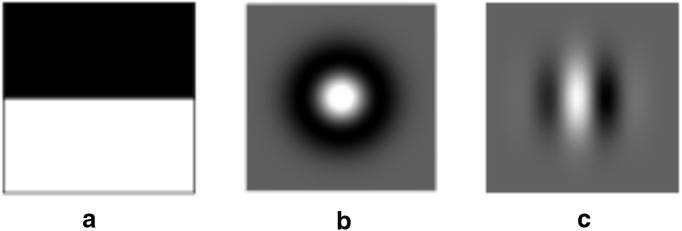
Illustration of receptive fields implemented in the visual input item in Neuronify. The user may choose between these and use them in combination with input from the camera on their device to simulate a neuron with a visual receptive field. ***A***, Rectangular edge-detecting receptive field. ***B***, Circular center-surround receptive field. ***C***, Orientation-selective receptive field.

Visual input is a spike generator based on visual input from a camera connected to the user’s device. This mimics a neuron with a visual receptive field (Dayan and Abbott, 2005; Mallot, 2013). There are three types of receptive fields implemented in Neuronify. (1) Rectangular edge-detecting. This edge-detecting receptive field consists of two adjacent rectangular ON and OFF regions of the same size. The orientation of the ON and OFF region can be adjusted. This field is shown in Figure 5*A*. (2) Circular center-surround. The field is defined as the difference of two Gaussian functions, a type of receptive field found in the retina and lateral geniculate nucleus (Rodieck and Stone, 1965; Hoffmann et al., 1972). The center type (ON-center or OFF-center) can be set in the setting menu. This field is shown in Figure 5*B*. (3) Orientation-selective. The field is defined as a Gabor function, a type of receptive field found in the primary visual cortex (Hubel and Wiesel, 1962). The orientation of the field can be adjusted in the setting menu. This field is shown in Figure 5*C*.

In reality, receptive field neurons have a temporal component such that the response depends not only on the present visual stimulus, but also the stimulus in the recent past (Dayan and Abbott, 2005). In Neuronify, however, the visual input item currently depends only on the instantaneous input.

### Sensors

To measure the activity of neurons, Neuronify provides several measurement items: a voltmeter, a spike detector, a firing-rate plot, and a loudspeaker.

#### Voltmeter

This item can be used to record the membrane potential from one or more neurons. The plot range can be adjusted by the user. When the voltmeter is connected to more than one neuron, it will be shown as rows of voltage traces, where the traces are identified by the labels of the neurons.

#### Spike detector

This item shows spikes from one or more neurons. When the spike detector is connected to more than one neuron, the spike times are shown in rows, where each row corresponds to one neuron. If a neuron has a label, it will be shown in the spike detector. The time range (window) can be adjusted by the user.

#### Firing rate

This item shows the firing rate measured in spikes per second of one or more neurons. When connected to more than one neuron, the mean population firing rate is shown (averaged over connected neurons). The rate is calculated on-the-fly using a Gaussian window, where the window width can be adjusted by the user. The minimum and maximum firing rate shown in the plot can also be adjusted.

#### Loudspeaker

This item plays a sound when a connected neuron fires. The loudspeaker is able to play different sounds that can be chosen by the user. A loudspeaker can be connected to multiple neurons. In this case, the loudspeaker plays the sound every time any of the connected neurons fire.

### Technical aspects

Neuronify is developed using the cross-platform framework Qt (Qt developers, 2016) and is written in a combination of C++, QML, and Javascript. C++ is a programming language suitable for high-performance computations, while QML is a programming language for defining visual items in a graphical user interface.

In the following section, we will briefly discuss how to install Neuronify and the implementation details of the app. While this is a brief introduction, detailed information can be found online.

#### Installation

Neuronify is available to download for multiple platforms. The app can be found in the app stores for Android and iOS. For Ubuntu, Neuronify is available as a download in Ubuntu Software. For Windows and Mac, Neuronify is available as a zip file and a dmg image, respectively. While the app is only supported on the above platforms, it should compile and run on any platform supported by the Qt framework. This includes a number of desktop and mobile platforms, in addition to embedded devices. For installation on other platforms or if you intend to make modifications to the source code, please see the next section about building from source.

#### Building from source

Neuronify is open-source software, allowing users to download the source code and make changes to the app. For details about the open-source license, please see the LICENSE file that comes with your copy of the source code. The source code is made available online at http://ovilab.net/neuronify. To obtain the source code, you may either clone the repository using git or download the most recent release as a zip file.

Up-to-date installation instructions can be found in the README.md file in your copy of the source code. Neuronify requires a recent version of Qt to be installed. As of writing, the source code is compatible with Qt 5.7. Once Qt is installed, the file neuronify.pro can be opened in Qt Creator, from which it can be built and run.

#### Architecture

Neuronify has a main engine named GraphEngine. This manages all the items in the simulation and is defined in the C++ class of the same name. The neurons and other items are structured within GraphEngine as nodes in a graph, hence the name. Each connection (or synapse) is handled as an edge in this graph. The behavior of an item or edge is defined by its implementation of certain functions. The most notable functions are stepped, fired, and receivedFire. These functions can be overloaded in either C++ or QML for new items. This flexibility allows for fast prototyping of items in QML while the final implementation can be written in C++ for improved performance.

In addition to fast prototyping, we have made this choice of architecture to allow for a future collaborative feature where the user can share custom items and neuron models with each other. This will be a feature in a future version of Neuronify.

The GraphEngine class is written in C++ and keeps track of all the nodes and edges in the simulation. The nodes are items such as cells and current generators, while the edges are synapses connecting the items. The GraphEngine class is responsible for moving the simulation forward by calling on all nodes and edges to do a time step. This stepping solves the coupled ordinary differential equations for all the cells and synapses. If a cell fires during the time step, it reports this to the GraphEngine, which passes on this information to any connected cells in the next time step.

The visual representation of items is defined in QML, a programming language made specifically for the Qt application framework. QML is declarative, which means that the programmer defines logical expressions rather than a sequence of operations. This makes it a good choice for programming graphical items and prototyping neuron models. Dynamic items are defined by their engine. They are implemented by defining the onFired, onStepped, and onReceivedFire signals.

The RegularSpikeGenerator is an example of such a dynamic item which is implemented in QML. It generates a spike with a constant interval, much like a metronome. We defined onStepped to sum up the time since last it fired. If the time is more than the interval, we call the fire function. Once it has fired, we set the time since last firing to zero. Below is a simplified definition of the RegularSpikeGenerator’s engine in QML:

NodeEngine {

property real rate

property real timeSinceFiring

//here we have omitted functions and

//properties for initialization and saving

onStepped: {

timeSinceFiring += dt

if(timeSinceFiring > 1.0/rate) {

fire()

timeSinceFiring = 0.0

}

}

}

While most items are best defined by these functions directly, Neuron objects share many common properties and are therefore possible to define using a specialized engine named NeuronEngine. This engine can have Current objects as children. The current property of each Current object is summed by the engine at each time step. This sum, together with the synaptic and injected currents, defines the total current over the neuron’s membrane. The NeuronEngine automatically controls firing by keeping track of the neuron’s voltage. Whenever the voltage goes above the firing threshold, the neuron will fire. In addition, the NeuronEngine adds any synaptic input current to the user-defined currents. A user that wants to implement a custom neuron model therefore only needs to define the currents of this model. Below is an example of a QML implementation of a NeuronEngine that defines a leak current:

NeuronEngine {

id: engine

Current {

id: leakCurrent

property real resistance: 100.0e6//ohm

onStepped: {

var Em = neuronEngine.restingPotential

var V = neuronEngine.voltage

var R = resistance

var I = -1.0/R * (V - Em)

current = I

}

}

}

Currently, neuron models are added by modifying the source code of Neuronify. In coming versions of Neuronify, we will add the possibility to develop new models as plugins. Finally, with the addition of collaborative features, we will make it easy to share these models with others.

#### File format

The saved simulations are stored in the JSON file format. This allows the use of the Javascript functions JSON.stringify() and JSON.parse() to serialize and deserialize the items, respectively. Because the JSON.stringify() function would include all properties of a QML item, although not all are interesting to save, we have included a custom class called PropertyGroup that contains QML aliases for all the properties to save. This is stored in a list named savedProperties on each item. When we iterate all the items that are to be saved, we find all PropertyGroups in savedProperties and run JSON.stringify() on these. This turned out to be a very powerful way to add saved properties for new items.

To enable the above defined neuron for saving, we need to add the resistance property. The other properties that already exist on NeuronEngine are already enabled for saving by default. We add the resistance property in the following way:

NeuronEngine {

id: engine

savedProperties: PropertyGroup {

property alias resistance: leakCurrent.resistance

}

Current {

id: leakCurrent

//definition of leakCurrent as above

}

}

Once saved, all nodes and edges of the GraphEngine are gathered in the JSON file. In addition, the current version of the file format is saved to ensure the file is read back correctly if the file format has changed. The main structure of a saved file looks like this:

{

"fileFormatVersion": 3,

"edges": [

…

],

"nodes": [

…

],

"workspace": {

…

}

}

Here, we have omitted the contents of nodes and edges and the workspace properties for brevity.

## Results

Here, we present some examples of neural network motifs that can be created with Neuronify. The below four examples can be found in Neuronify together with other premade simulations.

### Textbook example of spike threshold

The large variety of networks that can be built with Neuronify opens up the possibility to use the app in neuroscience courses. One possible use is as an interactive alternative or addition to traditional illustrations. To illustrate this ability of Neuronify, we have reproduced figure 8.5 from Sterratt et al. (2011).

**Figure 6.**
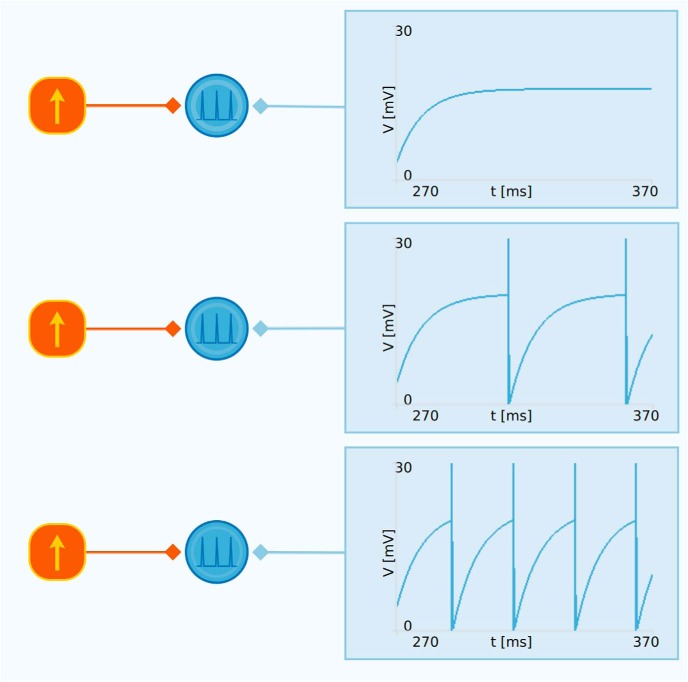
Example of how Neuronify can be used to create interactive illustrations for neuroscience courses. This is a reproduction of figure 8.5 in Sterratt et al., 2011. The example shows how different levels of current injection into a neuron model results in different firing rates. Note that this example uses an artificial resting potential of 0 mV.

This example demonstrates how different levels of current injected into a neuron produce different behavior and firing rates. As shown in Figure 6, there are three cases in this example, one which results in no firing, one with a low firing rate, and one with a higher firing rate. It is observed that the level of current injection must be sufficiently high to bring the membrane potential to the firing threshold, otherwise the cell will not fire at all. For currents above the threshold for firing, increased levels of current injection will result in higher firing rates.

The benefit of an interactive example when teaching is that the student, at will, can adjust the level of current injection and properties of the neuron model to explore how this changes the dynamics. These changes are presented in real time to the user, which is better than static illustrations or even figures produced with computational tools where the results are only available once the simulation is completed. With Neuronify, the results are instead immediately accessible to the user.

### Integration of synaptic inputs

**Figure 7.**
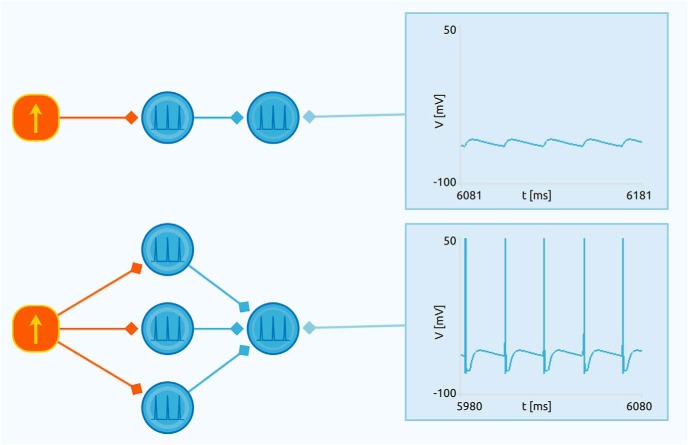
Example illustrating integration of synaptic inputs. In the upper circuit, the output neuron only receives input from a single presynaptic neuron. This input alone is not sufficient to make the output neuron spike. In the lower circuit, the output neuron instead receives input from three presynaptic neurons. This makes the neuron fire, thus illustrating how a neuron effectively integrates the synaptic input it receives to produce spikes. In the app, this example uses touch sensors instead of a current source for a more interactive illustration of this behavior.

Most neurons receive synapses from many neurons and require more than one synaptic input to reach threshold and fire. This summation, i.e., integration, of synaptic inputs determines the firing of neurons, and the principle is illustrated by the example in Figure 7.

### Feedback inhibition

**Figure 8.**
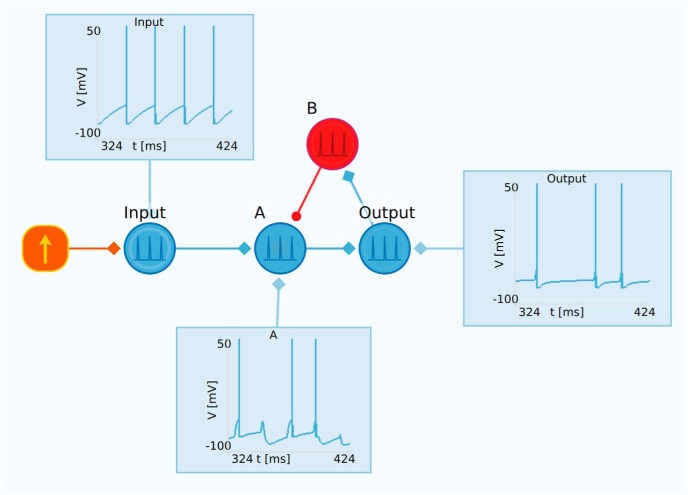
Example of gain control with feedback inhibition. The input neuron receives a constant direct current input and is connected to neuron A, which in turn is connected to the output neuron. The output neuron is further connected to the inhibitory neuron B. Neuron B inhibits neuron A, which in total results in feedback inhibition, i.e., reduced activity in the output neuron compared with the input neuron.

Feedback inhibition is a key network motif that, for example, may provide gain control in brain circuits. An example of a network with feedback inhibition is shown in Figure 8. Here, a DC current source delivers a constant current to the excitatory neuron labeled “Input”. This neuron is connected to the excitatory neuron A, which again is connected to the neuron labeled “Output”. The output neuron is further connected to the inhibitory neuron B, which finally inhibits neuron A. The overall result is reduced activity both in neuron A and in the output neuron in comparison to the input neuron.

### Direction-selective network

**Figure 9.**
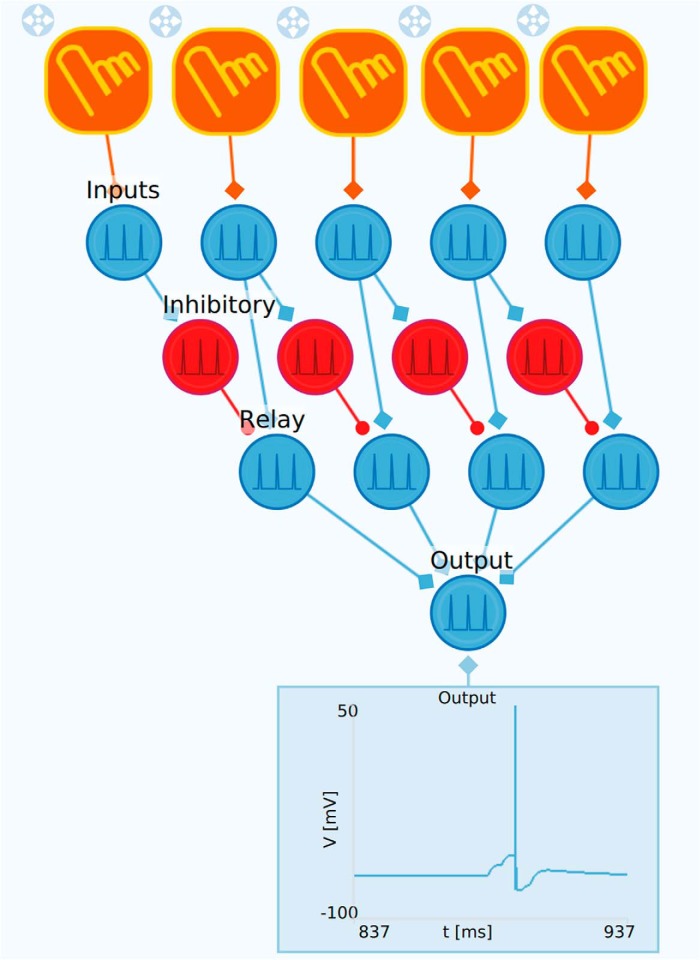
Example of direction-selective network. This example illustrates a direction-selective feedforward network based on one-sided lateral inhibitory connections. The upper row of touch inputs are connected to the input neurons. These are both connected to the relay neurons and the inhibitory neurons. Each inhibitory neuron inhibit the relay neuron positioned immediately to the right in the network. The relay neurons are connected to the output neuron. The effect of the inhibition is that the network only responds to input where the touch sensors are pressed sequentially from right to left but not in the opposite direction.

Direction-selective neurons are common in the visual system (Cruz-Martin et al., 2014; Liu, 2015) and are expectedly involved in motion detection. One way to create networks with direction-selective neurons is to use feedforward inhibitory connections with lateral connections in one direction only. An example is shown in Figure 9. Here, we have a linear array of input neurons that receive inputs from touch sensors and converge onto a single output neuron through “one-sided” lateral inhibition. The output neuron will respond to a sequential set of touch signals from right to left, but not in the opposite direction. This stems from the network design where the inhibitory neurons provides feedforward inhibition only to the relay neuron placed to the right in the network. Thus, if the sequential touch signal goes from left to right, the relay neurons will already be inhibited when the excitation arrives from the input layer. Therefore, no relay-neuron spikes, and consequently, no spikes in the output neuron, will be generated. However, with a touch sequence from right to left, the inhibition arrives too late to prevent the firing of the relay neurons and the output neuron.

## Discussion

In this article, we have presented Neuronify, an educational app that provides an interactive way of learning about neurons and neural networks. In Neuronify the user can add neurons, current sources, spike generators, and sensory input devices to the workspace. This makes it possible for students to create and explore their own neural networks without the need for programming. Students can build intuition for complicated circuits and behavior of neural networks. Neuronify should be a useful tool in many neuroscience courses because a large number of phenomena and networks can be demonstrated with the app. An additional use of Neuronify is as a proof-of-concept software, where the user easily can test the behavior of a simple network before implementing a more complex version in a suitable tool.

We plan to introduce more features in Neuronify in the future. One obvious candidate is synaptic plasticity. In short-term synaptic plasticity (Tsodyks and Markram, 1997), the synaptic efficacy is transiently changed, with typical time constants of less than a second, depending on the detailed pattern of afferent spike trains. In long-term plasticity, long-lasting changes in the strength of synaptic connections are induced, either long-term potentiation (LTP) (Bliss and Lomo, 1973) or long-term depression (LTD) (Ito, 1989). Inclusion of such plasticity into Neuronify would require detection of specific firing patterns and a modification of the synaptic strengths according to specific rules when various spike patterns are detected (Sterratt et al., 2011). While the inclusion of LTP and LTD would be particularly exciting as it would allow the user to create networks that can “learn,” a challenge would lie in visualising synaptic dynamics intuitively. It must be easy for the user to see the change in synaptic strength in addition to the change in network behavior.

New types of neurons can be implemented to explore a wide class of networks such as Izhikevich neurons (Izhikevich, 2003), adaptive-exponential integrate-and-fire neurons (Brette and Gerstner, 2005), or Hodgkin-Huxley type models (Sterratt et al., 2011).

Note that the list of possible new features to include in Neuronify is not exhaustive, nor a guarantee that they will be implemented. Exactly which features will be implemented depends on the feedback we receive.

Online sharing of user-generated networks and items is planned for a future version of Neuronify to foster a community of Neuronify users. This could inspire creativity and allow users to easily search and find networks of interest. It would also be a place where networks specific to a neuroscience course could be uploaded and shared with students.

We are hopeful that Neuronify can be valuable tool in neuroscience courses around the world and even inspire the creation of other educational tools in neuroscience.

*Note Added in Proof:* The text “Neuronify is available from http://ovilab.net/neuronify” was accidentally left out of the significance statement in the version of this article that was published on-line on March 9, 2017, as an Early Release Article. Further, a link to the source code was accidentally left out of the “Building from source” section. This has since been corrected.

## Synthesis

Reviewing Editor: Upinder Bhalla, National Center for Biological Sciences Decisions are customarily a result of the Reviewing Editor and the peer reviewers coming together and discussing their recommendations until a consensus is reached. When revisions are invited, a fact-based synthesis statement explaining their decision and outlining what is needed to prepare a revision will be listed below. The following reviewer(s) agreed to reveal their identity: Andrew Davison

SYNTHESIS

The reviewers both appreciated the quality of the manuscript and the software. The authors should refer to previous educational tools for neuroscience and explain how Neuronify is an improvement on the state of the art.

REVIEWER'S COMMENTS

Reviewer 1:

...

One minor point for revision: the manuscript does not reference any prior educational software tools for neuroscience. The abstract states that "few educational apps are available in neuroscience" - if there are so few, they could all be cited in the Introduction.

Typo in the legend for Fig. 2: "connected to a excitatory neurons"

Reviewer 2:

...This manuscript describes Neuronify, an online and mobile application which can be used to teach key concepts in neuroscience. The manuscript is well written and the motivation for and the key features of the app are well presented. It is good that the application is open source and freely available to all, and that community feedback and contribution is welcome. The examples included, while simplified, will provide a solid introduction to simulating neuronal circuits for someone with no computational experience.

There is not much discussion on previous work in the area of (online) educational tutorials for (computational) neuroscience, even though this is by no means a new area. More examples should be added of previous efforts, e.g. there have been many HH based tutorials, for example https://neuromembrane.ualberta.ca. Other standalone applications for creating neuronal networks through graphical interfaces should also be discussed briefly, e.g. Spinecreator, emergent.

Minor points:

Section 2.3 the distance between -> the difference between

s in equation 5 should be explained

Since we with current based synapses risk -> since with current based synapses we risk...
